# PFKFB3 Increases IL-1*β* and TNF-*α* in Intestinal Epithelial Cells to Promote Tumorigenesis in Colitis-Associated Colorectal Cancer

**DOI:** 10.1155/2022/6367437

**Published:** 2022-08-16

**Authors:** Hongbin Yu, Chuang Dai, Wei Zhu, Yude Jin, Chunhui Wang

**Affiliations:** Department of General Surgery, First People's Hospital Affiliated to Huzhou Normal College, Huzhou, China

## Abstract

Colorectal cancer (CRC) is significantly correlated with inflammatory bowel disease, which usually manifests as chronic relapsing-remitting colitis. Phosphofructo-2-kinase/fructose-2,6-biophosphatase 3 (PFKFB3) can catalyze to produce fructose-2,6-bisphosphate and function as an oncogene. In this study, we revealed the function of PFKFB3 in colitis-associated CRC (CAC) and the potential mechanism. RT-qPCR and Western blot were utilized to detect the level of PFKFB3 expression. Increased PFKFB3 expression was observed in the mouse CAC model and CAC patient samples. We identified that overexpression of PFKFB3 in intestinal epithelial cells (IECs) could increase the proliferation, migration, and invasion of CRC cells by the coculture system. Mechanistically, overexpression of PFKFB3 induced phospho-p65 and promoted the expression of IL-1*β* and tumor necrosis factor alpha (TNF-*α*) in the development of colitis and CAC. In addition, PFK158, the PFKFB3 inhibitor, could reduce the CRC cell viability, migration, and invasion caused by PFKFB3 overexpression. In conclusion, overexpression of PFKFB3 promoted tumorigenesis in CAC by inducing phospho-p65 and expression of IL-1*β* and TNF-*α*. Our study suggested that PFKFB3 acted as a potential treatment target for CAC.

## 1. Introduction

Colorectal cancer (CRC) is becoming the third most common cancer in the world with high mortality [1]. Multistep processes are involved in colorectal carcinogenesis, including a serrated pathway, carcinoma sequential pathway, and inflammatory pathway [2]. The mechanism of the inflammatory pathway is that the inflammation process could promote cell mutation and accelerate the cycle of wounding and repair in epithelial cells, resulting in colitis-associated cancer (CAC) [3, 4].

During the pathophysiological process of CAC, the immune cells were infiltrated and the proinflammatory and anti-inflammatory cytokines secretion are imbalanced. The single cell analysis of IBD patient tissues indicated that *T* and B lymphocytes, activated dendritic cells, and macrophages consist of the network in the inflammatory process [5]. It demonstrated that macrophages could secrete a range of proinflammatory cytokines during the progression of CAC, such as IL-1*α*, IL-1*β*, and tumor necrosis factor alpha (TNF-*α*). However, it has also been reported that intestinal epithelial cells expressed immunomodulatory cytokines during active ulcerative colitis and Crohn's disease. These signal pathways could activate intestinal epithelial cells (IECs) activity to induce a microenvironment transformation to develop tumor formation. It indicated that LPS-induced epithelial barrier dysfunction could be abolished by DNMT3a silencing or TNFSF13 overexpression, as well as abrogated the effect of IEC-regulated B cell differentiation [6], which indicated that LPS could induce epithelial dysfunction.

PFKFB3 (phosphofructo-2-kinase/fructose-2,6-biophosphatase 3) is an enzyme to produce fructose-2,6-bisphosphate (F-2,6-BP), involved in glycolytic activation [7]. PFKFB3 has been identified in many cancers, including breast cancer [8], pancreatic cancer [9], and gastric cancer [10]. PFKFB3 can promote cell proliferation through upregulation of cyclin-dependent kinase-1 (CDK1) and p27 [11]. Although the function of PFKFB3 in CRC cell lines has been demonstrated [12], the function of PFKFB3 in CAC remains unclear. NF-*κ*B plays a crucial role in inflammatory responses via regulating the synthesis and release of cytokines/chemokines, like tumor necrosis factor (TNF)-*α* and interleukin (IL)-1*β*, to promote the inflammatory responses. PFKFB3 could regulate endothelial and myocardial inflammation through the NF-*κ*B signaling pathway [13]. From our study, the expression of PFKFB3 level in IECs was increased to exacerbate tumorigenesis. We identified overexpression of PFKFB3 in IECs could increase proliferation, migration, and invasion of CRC cells by the coculture system. Mechanistically, overexpression of PFKFB3 induced phospho-p65 and promoted the expression of IL-1*β* and TNF-*α* in the development of colitis and CAC.

## 2. Materials and Methods

### 2.1. Clinical Samples and Animal Study

A total of 20 samples were collected from patients with CAC who accepted surgery. Fresh tumor and adjacent nontumor tissue samples were blindly collected. All patients signed the consent form. The study was approved by the Ethics Committee of First People's Hospital affiliated to Huzhou Normal College.

Eight weeks old C57BL/6 mice were injected with azoxymethane intraperitoneally and treated with water containing 1.5% dextran sulfate sodium (DSS) and with regular water for 2 weeks. All animal experiments were performed according to the National Institutes of Health guide for the care and use of laboratory animals (NIH Publications, revised 1978). The study was approved by the Institutional Animal Care and Use Committee of Huzhou Normal College.

### 2.2. Cell Culture and Cell Transfection

CRC cell lines (Caco2) and HIEC-6 cell lines were obtained from American Type Culture Collection (ATCC). All cells were cultured in DMEM (Invitrogen, USA) with 10% FBS, with a humidified atmosphere containing 5% CO_2_ at 37°C. The HIEC-6 cell lines were transfected with pcDNA-PFKFB3 (Genewiz, China) and siRNA-PFKFB3 (GenePharma, China) by using Lipo 3000 (Thermo, USA). siRNA-PFKFB3 sequence: CGCAGCAAGCAUGGCAGAAU.

### 2.3. CCK-8 Assay

The treated cell was digested and added with a density of 10,000 cells per well added into the top chambers of transwell inserts with FBS-free DMEM. Two days later, the cell counting kit-8 (CCK-8) reagent was added in the bottom chambers and incubated for 2 hours. We determined the optical density (OD) at 450 nm using a multimode microplate reader.

### 2.4. Cell Invasion Assay

The treated cells were digested and added into the top chambers of transwell inserts with FBS-free DMEM. The cell density is 2 × 10^5^ cells per well. DMEM with 10% FBS was added to the bottom chambers. After 6 hours, 4% paraformaldehyde was used to fix the inserts, and 0.1% crystal violet solution was used to stain cells. The images were obtained under a microscope.

### 2.5. Wound Healing Assay

The treated cells were digested and cultured in 6-well plates. After coculture, a scratch was made with a 200 *μ*L pipette tip. The debris was washed with PBS, and cell was incubated with DMEM for another 12 hours. At least 3 random areas were photographed to assess the closure of the gap.

### 2.6. RNA Extraction and RT-qPCR

TRIzol (Takara, Japan) reagent was utilized to isolate the RNA in cells and tissue. The SuperScript™ RT reagent kit (Takara, Japan) was used. Total RNA was used to reserve to synthesise cDNA templates. Expression of the mRNAs was detected with SYBR green according to the standard protocol. The primer sequences were listed: PFKFB3 forward TTGGCGTCCCCACAAAAGT, reverse AGTTGTAGGAGCTGTACTGCTT; IL-1*β* forward ATGATGGCTTATTACAGTGGCAA, reverse CGTCGGAGATTCGTAGCTGGA; TNF-*α* forward ATGACACCACCTGAACGTCTC, reverse CTCTCCAGAGCAGTGAGTTCT; GAPDH forward TGGATTTGGACGCATTGGTC, reverse TTTGCACTGGT ACGTGTTGAT.

### 2.7. Western Blotting

Proteins were isolated by RIPA buffer containing phosphatase and protease inhibitors (Roche, US). Equal total proteins were separated by SDS/PAGE gels and blotted onto PVDF membranes (Millipore, USA), followed by 5% milk blocking. The membranes were incubated overnight at 4°C with anti-phospho-NF-*κ*B p65 antibody (#3033; Cell Signaling Technology), anti-NF-*κ*B p65 antibody (#8242; Cell Signaling Technology), anti-PFKFB3 antibody (#13123S; Cell Signaling Technology), or anti-GAPDH antibody (60004-1-Ig; Proteintech). Finally, the blot was observed via the ECL detection system.

### 2.8. Immunofluorescence Assay

For p65 staining, the treated cells were washed with PBS and treated with 0.1% Triton *X* (Beyotime, China). Subsequently, blocking buffer was added and the primary antibody was incubated overnight at 4°C. Then, cells were treated with secondary antibody and DAPI. Images were captured by a microscope.

### 2.9. Statistical Analysis

All measurements were presented as the mean ± standard deviation (SD) from three independent experiments. Differences were determined using a two-way analysis of variance (ANOVA) or unpaired Student's *t*-test by Prism software. Statistical significance was defined as a *P* value < 0.05.

## 3. Results

### 3.1. PFKFB3 Expression Is Increased during Colitis and Colorectal Tumorigenesis

**Figure 1:**
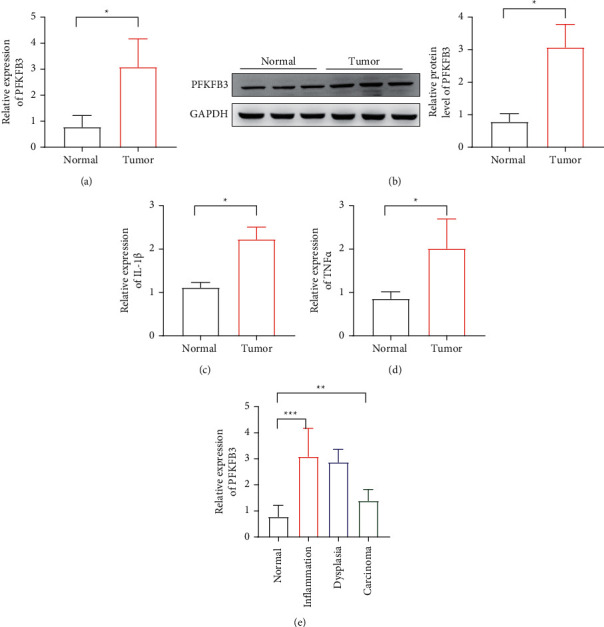
The level of PFKFB3 expression in CAC. (a) The expression of PFKFB3 mRNA in CRC tumor. (b) The protein level of PFKFB3 in CRC tumor. (c)-(d) The level of IL-1*β* and TNF-*α* detected in tumor by RT-qPCR. (e) Relative expression of PFKFB3 in colonic tissues from AOM/DSS-treated mice. ^*∗*^*P* < 0.05, ^*∗∗*^*p* < 0.01, ^*∗∗∗*^*p* < 0.001.

In order to detect the expression of PFKFB3 in colitis-associated cancer, we used RT-qPCR and Western blot to observe the PFKFB3 level in colitis-associated cancer (CAC) patient samples. It demonstrated that PFKFB3 expression was upregulated in CAC (Figures 1(a) and 1(b)). Additionally, the level of IL-1*β* and TNF-*α* was also increased in colitis-associated cancer (Figures 1(c) and 1(d)). Then, a mouse CAC model was established to detect the level of PFKFB3, and significantly increased PFKFB3 expression was detected in the inflamed, dysplastic, and carcinoma tissues (Figure 1(e)).

### 3.2. PFKFB3 Overexpression in IECs Exacerbates CAC Development

**Figure 2:**
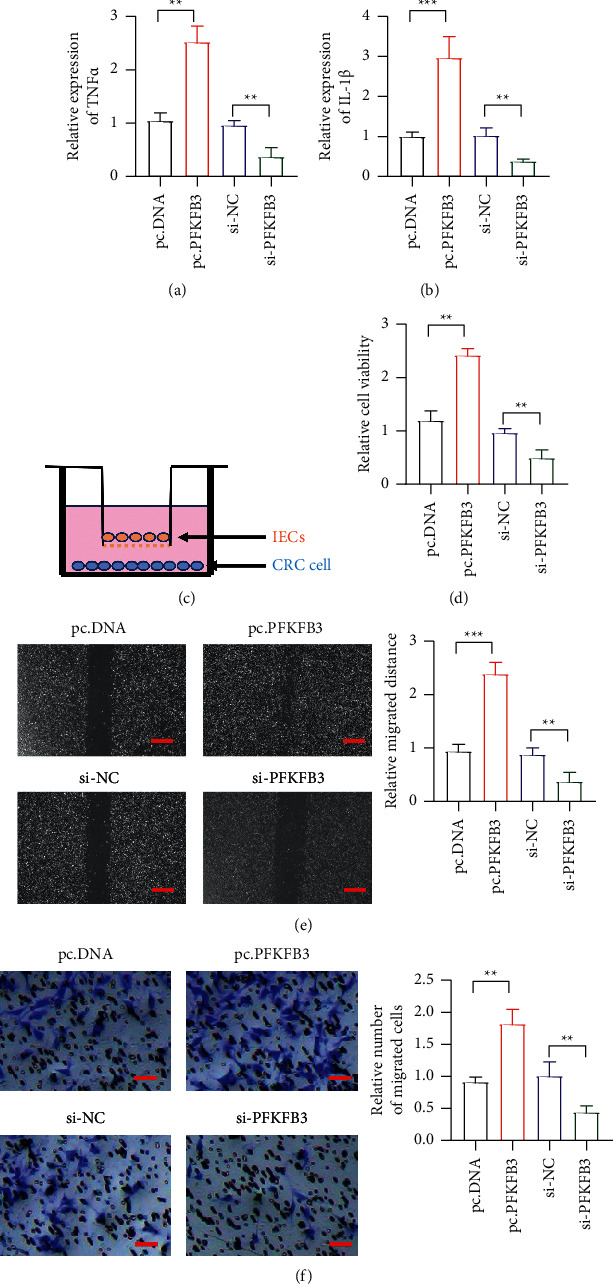
The role of PFKFB3 in CAC. (a)-(b) The expression of IL-1*β* and TNF-*α* level detected in PFKFB3 overexpression and knockdown. (c) The coculture assay detecting the influence of IECs on CRC cell. (d) The proliferation of CRC by PFKFB3 overexpression and knockdown in IECs. (e) The migration of CRC by PFKFB3 overexpression and knockdown in IECs; scar bar = 100 *μ*m. (f) The invasion of CRC by PFKFB3 overexpression and knockdown in IECs; scar bar = 50 *μ*m. ^*∗∗*^*P* < 0.01, ^*∗∗∗*^*p* < 0.001. pcDNA, empty vector; Si-NC, siRNA negative control.

As we know, IL-1*β* and TNF-*α* are important in the tumor microenvironment. First, we overexpressed or knocked down PFKFB3 in the IECs. It showed that overexpressed PFKFB3 could increase the expression of IL-1*β* and TNF-*α* (Figures 2(a) and 2(b)). Then, we established a coculture system to observe the effect of PFKFB3 in IECs on CRC cell lines (Figure 2(c)). We overexpressed or knocked down PFKFB3 in IECs and detected proliferation of CRC cell lines by CCK-8 cell lines. It showed that PFKFB3 overexpression in IECs could increase the proliferation ability of CRC cell lines (Figure 2(d)). We also detected the cell migration and invasion after PFKFB3 overexpression in IECs. The wound healing assay and transwell assay indicated that PFKFB3 overexpression in IECs could enhance the cell migration and invasion in CRC cell lines, while PFKFB3 knockdown could reduce the effect (Figures 2(e) and 2(f)). The results indicated that the PFKFB3 in IECs could induce the proliferation, migration, and invasion ability of CRC cell lines.

### 3.3. PFKFB3 Activated the NF-*κ*B Signal Pathway to Induce IL-1*β* and TNF-*α* in IECs

**Figure 3:**
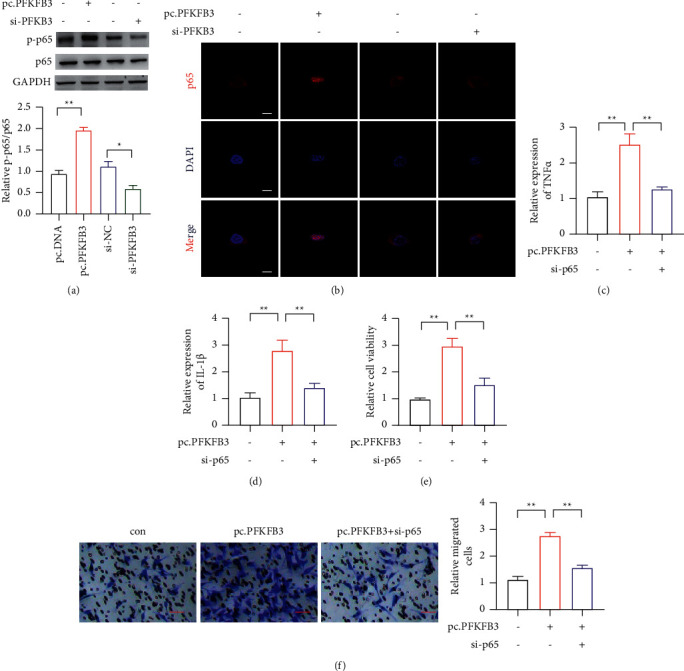
The p65 activation in CAC. (a) The phosphorylation of p65 detected in PFKFB3 overexpression and knockdown. (b) The nuclear translocation of p65 detected in PFKFB3 overexpression and knockdown; scar bar = 20 *μ*m. (c)-(d) The expression of IL-1*β* and TNF-*α* level by PFKFB3 overexpression and p65 knockdown in IECs. (e) The proliferation of CRC by PFKFB3 overexpression and p65 knockdown in IECs. (f) The invasion of CRC by PFKFB3 overexpression and p65 knockdown in IECs; scar bar = 50 *μ*m. ^*∗*^*P* < 0.05, ^*∗∗*^*p* < 0.01.

We detected the signal pathway in the PFKFB3 overexpressed IECs. Previously, it demonstrated that overexpression of PFKFB3 could increase the phosphorylation of p65. So, we detected the level of p65 phosphorylation in IECs. It indicated that overexpressed PFKFB3 could upregulate the phosphorylation of p65, while PFKFB3 knockdown could reduce the effect (Figure 3(a)). Then, the immunofluorescence assay was performed to reveal the p65 nuclear translocation (Figure 3(b)). It also demonstrated that PFKFB3 overexpression could increase p65 nuclear translocation. It also showed that PFKFB3 overexpression increased the level of IL-1*β* and TNF-*α*. However, the knockdown of PFKFB3 decreased the IL-1*β* and TNF-*α* levels (Figures 3(c) and 3(d)). Then, we also knocked down p65 in PFKFB3 overexpressed cells. It indicated that PFKFB3 could promote the CRC cell proliferation, migration, and invasion through NF-KB activation (Figures 3(e) and 3(f)).

### 3.4. PFK158 Ameliorates CAC Development

**Figure 4:**
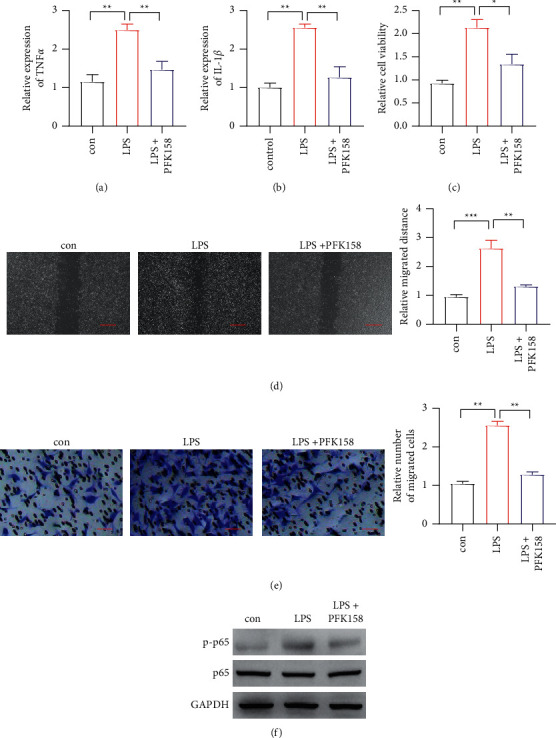
The PFK158 reduces cell proliferation, migration, and invasion. (a)-(b) The IL-1*β* and TNF-*α* expression level detected in PFK158 treatment. (c) The proliferation of CRC by LPS stimulation and PFK158 treatment in IECs. (d) The migration of CRC by LPS stimulation and PFK158 treatment in IECs; scar bar = 100 *μ*m. (e) The invasion of CRC by LPS stimulation and PFK158 treatment in IECs; scar bar = 50 *μ*m. (f) The p65 phosphorylation detected CRC by LPS stimulation and PFK158 treatment in IECs. ^*∗*^*P* < 0.05, ^*∗∗*^*p* < 0.01.

PFK158, a PFKFB3 inhibitor, was indicated to suppress the tumor development. Then, we used LPS to treat IECs, followed by PFK158 treatment. It showed that PFK158 could decrease the evaluated IL-1*β* and TNF-*α* caused by LPS induction (Figures 4(a) and 4(b)). Then, we used the coculture system to check cell proliferation, migration, and invasion of CRC cell lines. It observed that LPS-treated IECs could enhance cell proliferation, migration, and invasion of CRC cells, which was inhibited by PFK158 (Figures 4(c)–4(e)). Finally, we detected phosphorylation of p65, and it showed that PFK158 reduced phosphorylation of p65 caused by LPS treatment (Figure 4(f)).

## 4. Discussion

Nowadays, more and more attention has been paid to the immunity cells in the development of CAC, such as macrophages [14]. However, the tumor microenvironment consists of many types of cells. In our study, we focused on the role of IECs in the tumor microenvironment. It was observed that the PFKFB3 level was increased in colitis-associated cancer. The abnormal interaction between IECs and CRC is unclear, and an imbalance of inflammatory cytokines has not been demonstrated clearly in CAC progression. It showed that the level of IL-1*β* and TNF-*α* was also increased in colitis-associated cancer.

PFKFB3 functions as an oncogene to enhance the glycolytic activity for production of fructose-2,6-biphosphate, which activates 6-phosphofructo-1-kinase [15]. The role of PFKFB3 has been reported in many cancers, including breast cancer, pancreatic cancer, and gastric cancer. The role of PFKFB3 has been reported in CRC. However, its role in CAC remains unclear. We focus on the role of PFKFB3 in IECs to regulate the tumor microenvironment. The results indicated that PFKFB3 in IECs could induce proliferation, migration, and invasion ability of CRC cells. NF-*κ*B plays a crucial role in inflammatory responses via regulating the synthesis and release of cytokines/chemokines, like tumor necrosis factor (TNF)-*α* and interleukin (IL)-1*β*, to promote the inflammatory responses. PFKFB3 could regulate endothelial and myocardial inflammation through the NF-*κ*B signaling pathway. A recent study indicated that PFKFB3 overexpression could increase immune evasion and tumorigenesis in hepatocellular carcinoma by NF-*κ*B activation and enhance PDL1 expression [16] and could regulate the NF-*κ*B pathways in ovarian cancer [17]. So, we detected the level of p65 phosphorylation in IECs. It indicated that overexpressed PFKFB3 could upregulate phosphorylation of p65, while PFKFB3 knockdown could reduce the effect. Then, the immunofluorescence assay was performed to release p65 nuclear translocation. It also demonstrated that PFKFB3 overexpression could increase p65 nuclear translocation.

We further assessed the therapeutic function of PFKFB3. PFK158, the PFKFB3 inhibitor, has been approved by the FDA in clinical trials with pancreatic cancers and breast cancers, as well as many other cancers. It has been reported that PFK158 could decrease tumor growth in melanoma [18]. We used LPS to treat IECs, followed by PFK158 treatment. It showed that PFK158 could decrease the elevated IL-1*β* and TNF-*α* caused by LPS induction. The coculture system indicated that LPS-treated IECs could enhance cell proliferation, migration, and invasion of CRC cell lines through phosphorylation of p65, which was inhibited by PFK158. The results indicated that PFK158 was a potential candidate to treat CAC.

## 5. Conclusion

In conclusion, the level of PFKFB3 in intestinal epithelial cells (IECs) was increased to exacerbate tumorigenesis in mice. We identified overexpression of PFKFB3 in IECs could increase the viability, migration, and invasion of CRC cells by the coculture system. Mechanistically, overexpression of PFKFB3 induced phospho-p65 and promoted the expression of IL-1*β* and TNF-*α* in the development of colitis and CAC.

## Data Availability

The data used to support the findings of this study are included within the article.
